# The butterfly effect: collateral damage and impacts of antimicrobial strategies on the oral microbiome

**DOI:** 10.1038/s44259-025-00156-6

**Published:** 2025-10-01

**Authors:** Jason L. Brown, William Johnston, Mark C. Butcher, Mia Burleigh, Gordon Ramage

**Affiliations:** 1 Oral Sciences Research Group, Glasgow Dental School, School of Medicine, College of Medical, Veterinary and Life Sciences, University of Glasgow, Glasgow, UK; 2 Oral Biofilm Research Group, Safeguarding Health through Infection Prevention (SHIP), School of Health and Life Sciences, Glasgow Caledonian University, Glasgow, UK; 3 Division of Sport, Exercise and Health, Sport and Physical Activity Research Institute, School of Health and Life Sciences, University of the West of Scotland, Glasgow, UK

**Keywords:** Antibiotics, Antifungal agents, Antimicrobial resistance

## Abstract

The oral cavity is a complex environment hosting diverse microbial biofilms on different surfaces, all immersed in saliva that enables recolonisation and spread. These microbial populations fluctuate with diet, hygiene, antimicrobials, and disease. While biofilm control measures aim to protect health, they may cause unintended effects, including antimicrobial resistance (AMR). Persistent resistant microbes reshape oral and systemic niches through ecological disruption and genetic adaptation, which may negatively impact human health.

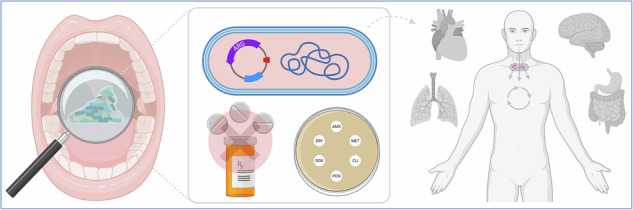

## Introduction

It is now well understood that the influence of antimicrobials, metabolites or new microorganisms, can have significant effects on the distribution of organisms within an ecological niche, promoting imbalance and leading ecological perturbations and the development of resistance^1,2^. This collateral damage, in terms of resistance in off-target populations, poses a significant and often unrecognised risk to patient health, particularly when these populations serve as reservoirs for future infections. This is especially the case for many critical drug-resistant pathogens. Morley and colleagues (2019) described the concept of ‘bystander’ organisms within an individual’s broader microbiome^3^. This can lead to depletion of resident microbiota, which in turn negatively impacts colonisation resistance. In addition, it can alter complex microbial communities that can affect how resistance can evolve. The lack of consideration of how the non-target microbe is impacted while attempting to deplete the perceived culprit pathogen is concerning. Researchers are now beginning to contemplate what impact that this collateral damage has to complex microbiomes.

The oral microbiome is believed to consist of over 700 species of bacteria, fungi, archaea and viruses^4–6^. In health, these microorganisms maintain a colonisation barrier, contribute to immunomodulation, and are involved in various beneficial processes, including the generation of nitric oxide^7–9^. In contrast, shifts within this microbiome are associated with a number of oral diseases, such as dental caries, gingivitis, and periodontitis^10,11^. The implications of these shifts also extend beyond the oral cavity, as numerous studies demonstrate an intricate link between oral pathobionts and systemic diseases^12–16^. Given that this has been extensively reviewed elsewhere^17,18^, we will instead focus on the overarching factors within the oral cavity that drive taxonomic changes within the oral cavity and beyond, through translocation of microorganisms to distal sites that may facilitate broader health impacts, such as antimicrobial resistance (AMR). The oral microbiome, whilst essential for oral and systemic health, is also a reservoir and transmission hub for antimicrobial resistance genes (ARGs): just a handful of genes that represent a functionally important component of the oral metagenome. Collectively known as the resistome, or antibiotic resistome to be exact^19^, microbial colonisation plays a critical role in shaping ARG dynamics^20^. This review considers the concept of the “butterfly effect”, a hypothesis in chaos theory that describes how small changes or events can have significant and far-reaching effects on complex systems. We consider how dietary factors, physical disruption, antiseptics and antimicrobials can have the capacity to induce off-target effects on the microbial communities through a series of small perturbations, ultimately leading to more impactful outcomes.

## Oral Resistome and the Diet

The resistome is present from infancy and alters throughout life in line with dietary influences, maturation events and lifestyle factors^21^. The initial establishment of the microbiome in infants has been shown to be heavily influenced by diet, with studies observing the potential for formula fed infants to exhibit a more cariogenic distribution of organisms when compared with breastfed infants^22^. Indeed, the fluctuation of plaque biomass and the effect on the oral microbiota is influenced by macronutrients, such as dietary sugars and fibre^23^. The drivers for the resistome in the oral cavity remain clear, but it is hypothesised that formula feeding may be a reason, as has been shown previously in the gut^24^. These ARGs were predominantly found in breastfed babies, leading to the hypothesis that these genes may have a protective function, rather than being of detriment to the host. Significant temporal changes in the resistome were identified in the first 2 years of life, then stabilising by 5 years. It is likely that indirect exposures to antibiotics (e.g., through food production/consumption) may also influence the mobilisation of ARGs. Similar to studies in the gut, high-sugar diets have been shown to influence the resistome^25^, and together these data indicate that diet influences the microbiome through induction of various stress responses.

Beyond infancy, the influence of dietary sugars has often been linked to the prevalence of known biofilm producing organisms within a reduced microbial diversity^26^, such as *Streptococcus mutans*^27,28^. It has been shown that *S. mutans* upregulates stress responses during fructose metabolism that are shared with hydrogen peroxide exposure^29^. Moreover, detailed analysis from a twin study revealed that the mobile element (*Tn916* – a family of transposases) was commonly identified in oral streptococci, which was associated with co-carriage of macrolide and tetracycline resistance^21^. This is suggestive that emergence of such ARG components in a particular niche may simply be a result of microbial shifts, from health to disease-states, leading to flourishment of higher burden of pathogens that harbour ARGs.

**Fig. 1:**
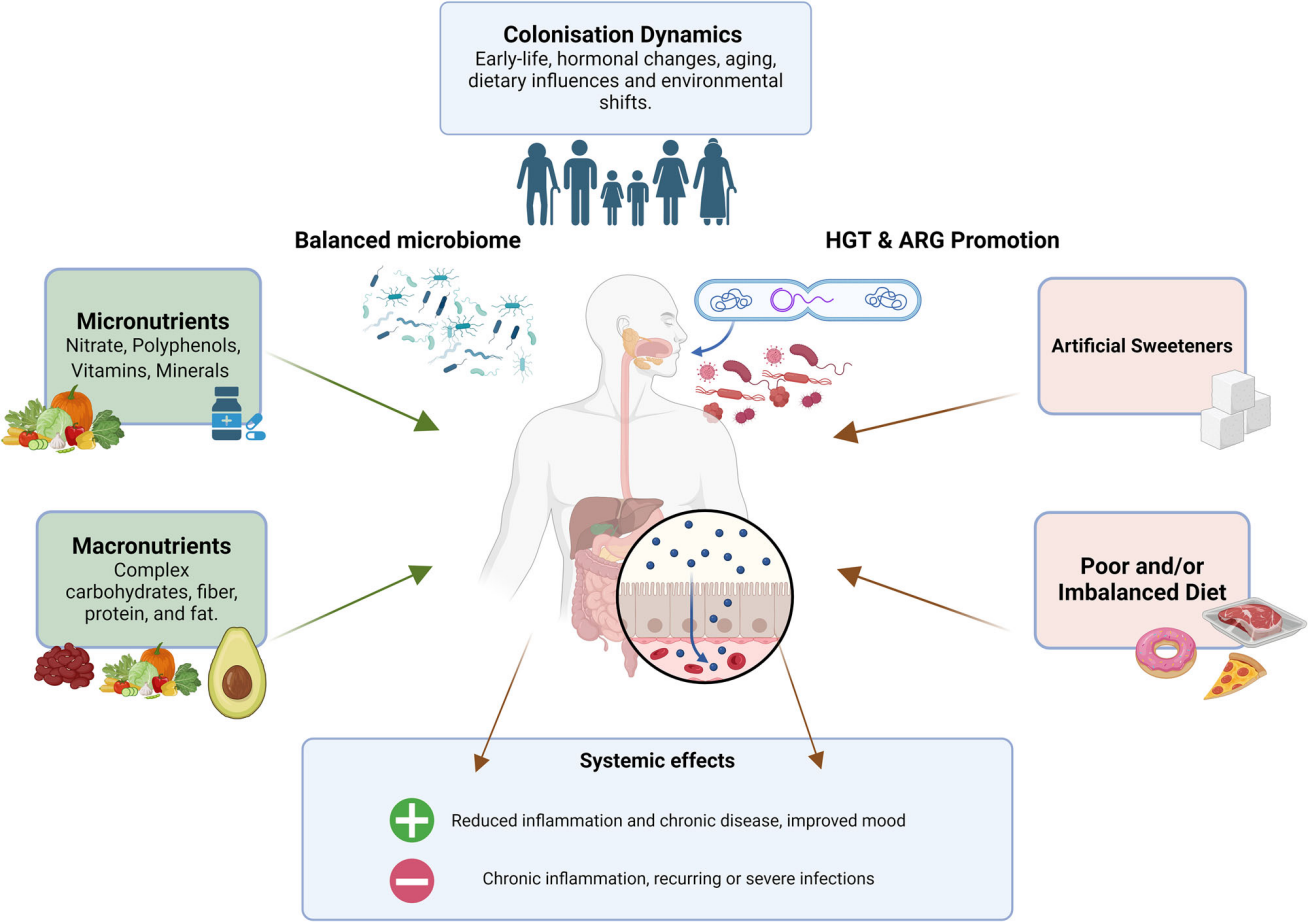
How dietary components, colonisation dynamics, and maturation events shape the oral microbiome and its role as a reservoir for ARGs. Micronutrients such as nitrate and polyphenols and macronutrients like fibre and healthy fats promote microbial stability, reducing potential for antimicrobial resistance gene (ARG) dissemination. Conversely, poor diets and artificial sweeteners create conditions which increase biofilm formation, horizontal gene transfer (HGT) and ARG mobilisation. These dietary influences impact systemic health, with balanced microbiomes reducing inflammation and supporting antimicrobial action, while poor diet exacerbates chronic inflammation. Figure created using BioRender.

Inversely, fibre-rich diets can promote beneficial commensals that may suppress resistance transfer potential^30^. Early microbial patterns affect susceptibility to horizontal gene transfer (HGT) of ARGs, which occurs predominantly in biofilms^31^. The diet continues to have profound effects on microbial colonisation throughout adulthood, with poor dietary choices and alteration of plaque ecology, disrupting homeostasis^32,33^. This has been shown to exacerbate resistance against antibiotics such as tetracyclines and macrolides, while promoting microbial translocation to distal sites, where ARGs can contribute and promote systemic infections^21^. These colonisation patterns underscore the importance of dietary interventions in maintaining microbial stability and mitigating ARG dissemination. This section outlines the intertwined nature of oral microbial ecology and systemic health and highlights that even small dietary changes can have far-reaching effects on host physiology and potential to influence ARG development. The interplay between colonisation dynamics, dietary components, and ARG dissemination is depicted in Fig. 1.

Micronutrients can also play a role in modulating colonisation dynamics and microbial activity in the oral cavity and thus will directly or indirectly affect ARG reservoirs. They are also well-studied for their roles in promoting oral and/or systemic health. Nitrate and nitrite are not traditionally considered micronutrients because they have negative health effects when consumed as food additives, for example, when added in their organic forms as a preservative for red meat and processed meats^34^. These sources typically make up 5% of nitrate and nitrite intake in people consuming an omnivore diet and are considered to be carcinogenic^35,36^. However, nitrate is also found in high quantities in leafy greens and root vegetables, with these foods being universally associated with systemic health benefits^35,37^. Regardless of the source, dietary nitrate must be reduced by species of oral bacteria, such as *Rothia*, *Neisseria*, *Actinomyces*, *Veillonella*, *Kingella* and *Propionibacterium*, into nitrite^7^. Nitrite is then further metabolised by nitrite-reducing bacteria into nitric oxide (NO) or ammonium, depending on the enzymatic pathways and environmental conditions^38,39^. Bacteria are essential to nitrate metabolism because mammalian cells are inefficient at this process^40–42^. NO is a multifunctional signalling molecule which is involved in various biological processes, such as host defence^43^, regulation of mucosal blood flow and mucus generation^44^, regulation of smooth muscle contraction^45^, cerebral blood flow^46^, glucose homeostasis^47^, and mitochondrial function^48^. In addition, NO exerts local antimicrobial effects by disrupting bacterial membranes and biofilm stability^49^. Evidence also shows that nitrate is a prebiotic which supports oral health by promoting the growth of health-associated species such as *Neisseria* and *Rothia*^50,51^ and helping to maintain a neutral pH even in the presence of acidogenic carbohydrates^52,53^.

Importantly, the use of antimicrobials such as chlorhexidine (CHX)-containing mouthwash impairs nitrate-reducing bacteria, potentially limiting these protective systemic effects and contributing to shifts in the microbial communities and a more acidic environment^40,54^. Despite being a critical tool for Dental Healthcare Practitioners in the management of oral pathologies, studies, like those produced by Bescos et al. ^54^ have demonstrated that CHX mouthwash alters microbial diversity, reducing beneficial species^54^. This has been confirmed recently, whereby CHX treatment of healthy individuals can promote a caries-associated bacterial community with increases in ARGs to antibiotics such as tetracycline^55^. Moreover, from a mechanistic standpoint Cieplik et al.^56^, in their review highlighted concerns that CHX may promote ARG selection through multiple mechanisms including, alterations to membrane composition, efflux pump expression, and ARG transfer in biofilms^56^. Indeed, there is evidence that CHX treatment can promote conjugation and spread of resistance genes in bacterial species^57,58^.

All of these changes discussed above have potential to create selective pressure towards ARG-dominant communities, including many other micronutrients^56,59^. Polyphenols, for example, are bound in the matrix of foods such as vegetables, roots, tea, berries, and red wine. Additionally, they can be consumed in purified forms via dietary supplements^60^. Combined consumption of polyphenols and NOC precursors (nitrate and nitrite) inhibits N-nitroso compound formation providing anticarcinogenic risk^61^. They are also reported to have positive effects on various other facets of systemic health including antioxidant, anti-allergic, anti-inflammatory, and antihypertensive effects^62^. In the oral cavity, they have been shown to have antimicrobial and immunomodulatory effects in periodontal disease^63^ and demonstrate anti-cariogenic properties^64–66^. In the context of resistance, polyphenols inhibit key bacterial enzymes and destabilise biofilms that harbour ARGs^67^, while their metabolites exhibit anti-inflammatory properties systemically (reviewed in Gupta and Birdi)^68^. This dual action highlights their potential to mitigate ARG dissemination and reduce chronic inflammation, which underpins many systemic conditions. Beyond nitrate and polyphenols, many other micronutrients contribute to immune function and mucosal integrity, which may indirectly influence colonisation and ARG dynamics. However, most dietary components are transient in the oral cavity meaning any potential influences are likely mediated through systemic effects rather than directly. Notably, oral exposure to dietary nitrate is sustained due to its integration in the entero-salivary circulation^69–73^.

Macronutrients have been well-studied in relation to their influence on gut microbial colonisation and ARG dynamics^30,74^. As touched upon previously, dietary fibre fosters beneficial colonisation through the production of short-chain fatty acids (SCFAs) via gut fermentation^75^. SCFAs reduce systemic inflammation and enhance mucosal immunity whereas mastication of tough dietary fibre will promote saliva stimulation, indirectly suppressing ARG transfer by facilitating a healthy oral environment. SCFAs have also been shown to inhibit *C. albicans* growth and stimulate the expression of Foxp3 and IL-17A in CD4 + T cells, however, excess SCFAs may also stimulate local oral cavity pathology development in addition to pro-inflammatory signalling, highlighting that there are site-specific complexities to consider in relation to SCFA and ARG development^76^. At present there is little evidence on the effect of protein on ARG, however, it is known to buffer pH through ammonia production^77^, and evidence suggests that specific amino acids may have antimicrobial effects. For example, salivary L-Arginine monohydrochloride has been shown to inhibit bacterial coaggregation in the oral cavity by decreasing the viscosity of extracellular polymeric substances produced by bacteria and altering cellular metabolism resulting in biofilm dispersion. Furthermore, cysteine reduces bacterial biofilm adherence and biofilm biomass^78,79^.

Functional additives in the form of non-nutritional sweeteners were first intended to replace cariogenic carbohydrates and promote oral health. Interestingly, natural sweeteners like xylitol also act as biofilm dispersal agents and have been proposed as co-therapies to aid the penetration of antimicrobials into the biofilm. Sucralose and aspartame disrupt microbial metabolic pathways and increase efflux pump expression^80^. Other non-nutritive sweeteners have also recently been associated with shifts in the gut microbiota, similar to those caused by antibiotics and therefore may promote the spread of ARGs in a similar manner. Single-cell sequencing has confirmed that saccharine, sucralose, aspartame, and acesulfame potassium enhance conjugation in bacteria and suggests that these compounds may increase the risk of resistance^81^.

Diet has the potential to shape host-microbe interactions, with implications for ARG dissemination, and systemic health. There is good evidence that nitrate and carbohydrates interact with bacterial species and have systemic effects, whereas, other dietary components and functional additives may indirectly influence ARG development through immune modulation or other host-microbe interactions. Conversely, it is worth noting that, whilst there is continued evidence of the links between diet and the oral health (and disease), it is important to contextualise these findings within broader population-level trends. Several large-scale population studies have demonstrated remarkable consistency in the composition of the oral microbiome across diverse geographic regions^82–86^, suggestive that other factors need careful consideration when studying the concept of the “oral resistome”. These observations indicate that while diet may influence oral health through short-term perturbations or in specific contexts (e.g., frequent sugar intake or micronutrient deficiency), such effects may be transient or secondary to geographical location, host-related factors (e.g., immune status, salivary flow) or treatment interventions. Indeed, the oral microbiome can often be viewed as a “stable ecosystem” in comparison to other ecological niches such as the gut (as reviewed in Joseph and Curtis, 2021^87^). Furthermore, while we have presented evidence here of the influence dietary decision making has on the oral microbiota, the clear influence of oral hygiene and treatment regimen on such communities cannot be understated. As oral healthcare has developed, the nuance of oral care has become highly varied and personalised, but much remains to be investigated on the potential impact of particular interventions^88^.

## Impacts of Physical Disruption

It is estimated that around 3.5 billion people suffer from oral disorders worldwide^68^. Despite these extreme numbers, one of the most important methods of plaque control in modern history continues to be physical removal (toothbrushing, interdental brushing and non-surgical therapies). The purpose of these approaches is to maintain a preponderance of immature plaque species (pioneers and mixtures of aerobes and facultative species), whilst preventing the community overgrowth of pathogens. Nevertheless, in a vast majority of the population, careful consideration to prevent of pathogenic plaque species occupying niches must be made. Obvious avenues to control this include toothpastes, which have been developed and improved with surfactants and/or antimicrobial agents which support microbial suppression, particularly of pathogens whilst shifting the oral microbiota and/or its metabolic pathways to one more closely associated with oral health^89–91^.

Some individuals remain prone to oral diseases despite consistent hygiene practices. Importantly, the mechanical removal of plaque is a non-selective process, and thus, whilst it helps reduce harmful taxa, it may also disturb beneficial members of the microbiota. In practice, however, oral health is typically preserved or restored because of the colonisation resistance exerted by commensal species. Still, in cases such as periodontitis, studies have shown that a dysbiotic signature remains within the oral microbiome following routine debridement, even 24 h after therapy^92^. It is well recognised that mechanical disruption of the oral biofilm can result in transient bacteraemia, particularly following invasive procedures such as scaling or root planing^70–72^. However, the vast majority of these episodes are short-lived and rapidly resolved by the host immune system^70,75^. Crucially, the risk of bacteraemia is not exclusive to professional mechanical plaque removal (PMPR). In fact, the accumulation of plaque and associated gingival inflammation increases the risk of spontaneous bacteraemia during routine actions such as chewing, toothbrushing and scaling^93^. Thus, regular plaque removal reduces the overall frequency and severity of bacteraemia in the long term by lowering microbial load and improving mucosal integrity. Nonetheless, in susceptible individuals, such as those with pre-existing heart valve abnormalities or immunosuppression, even transient bacteraemia can pose serious risks. It has been recognised for many years that infective endocarditis (IE) often results from oral microorganisms^76^, and recent guidelines from the European Society of Cardiology strongly recommend antibiotic prophylaxis for high-risk patients prior to invasive dental procedures^77^. Beyond IE, evidence also suggests that this bacteraemia may indirectly contribute to a range of systemic diseases. For example, oral bacteria have been found to colonise atherosclerotic plaques^12,78^, potentially promoting the progression of atherosclerosis^79^. Possible mechanisms behind this association have been recently described in reference^94^, believed to be driven by local dissemination of pathobionts (*Porphyromonas gingivalis, Aggregatibacter actinomycetemcomitans, Prevotella intermedia, Tannerella forsythia* and *Fusobacterium nucleatum*) and/or the related release of inflammatory mediators from the oral cavity. These pathobionts are often hallmarks of a dysbiotic microbiome, and their enrichment reflects an imbalance that may favour chronic inflammation and microbial translocation. Interestingly, a “Trojan Horse Approach” has also been proposed, whereby intracellular survival of these organisms may promote additional dissemination to distal sites^94,95^.

Another important, yet often overlooked, pathway for oral-systemic translocation is the respiratory tract^85^. The upper respiratory tract is directly connected to the oral cavity, and it is recognised that inhalation of oral bacteria contributes to aspiration pneumonia^86–89^. Accordingly, poor oral hygiene is a known risk factor for pneumonia^90^, and oral bacteria have been identified in lung aspirates of pneumonia patients^87,91^. The use of dental appliances, such as dentures, may further increase this risk, with dentures being associated with a higher likelihood of respiratory infection^85,92^. Supporting this association, a study examining over 100 dentures found that 64.6% were colonised by common respiratory pathogens, including *Staphylococcus aureus, Streptococcus pneumoniae, Streptococcus pyogenes, Pseudomonas aeruginosa, Haemophilus influenzae* type B, and *Moraxella catarrhalis*^93^. Moreover, in hospitalised patients with endotracheal tubes, the mixing of the oral microbiome with the respiratory microbiome can cause depletion of diversity and resident microbiota^94^. Indeed, within these unique microbiomes, novel intra- and inter- kingdom interactions exist^95^, which has implications for the development of thick and complex biofilms^96^.

## Controlling Plaque with Mouthwashes

There is mounting evidence that oral mouthwashes are important drivers of oral microbial dysbiosis: defined as a microbial imbalance resulting in overgrowth of the pathogenic community, leading to a deleterious impact on the host (e.g., the onset and progression of periodontitis or dental caries). Their blunderbuss approach is crude and untargeted, which has the consequence of indiscriminate killing that leads to microbiome destabilisation and primary or secondary disease progression rather than resolution^96^. There is also growing evidence that resistance can evolve to these over-the-counter (OTC) mouthwashes^55^. There are many of these to choose from, including sodium fluoride, hydrogen peroxide, cetylpryridium chloride, povidone iodine, alcohols, essential oils, and even propolis. Their impacts on the microbial in oral health and disease are extensively reviewed elsewhere (2023), though unarguably the mainstay and workhorse antiseptic within dentistry remains CHX^96^.

There is a significant body of evidence to imply that CHX (0.01% to 0.2%) is the ‘gold standard’ as an antiseptic for plaque control in patients deemed to need advanced oral hygiene advice or other chemotherapeutic intervention^97^. CHX is positively charged and interacts with the bacterial cell wall at low bacteriostatic concentrations, whereas at high bactericidal concentrations it leads to cell leakage and coagulation of the cytoplasmic components. The literature is littered with in vitro studies showing that CHX is effective against mono- and polymicrobial biofilms, and that despite its potency it can by cytotoxic^98^. Using defined biofilms from a 14 species in vitro model and an ex vivo tongue biofilm model it was shown that CHX treatment induced significant changes in microbiota composition and metabolic activity^99^. Despite leading to rapid kill, subsequent regrowth was evident with disease associated traits increased (e.g., higher abundance of pathobiont strains or metabolic shifts such as lactate). These data indicate that untargeted and indiscriminate killing leads to negative outcomes. This phenomenon is not limited to this study, with CHX treatment reported to cause significant decreases in saliva pH and a reduced capacity to buffer, with similar effects on saliva lactate and glucose levels reported in the aforementioned study^54^. Furthermore, in a study of 20 patients who applied a 0.2% CHX mouthwash twice daily for 4 weeks who had their saliva and supragingival plaque microbiota assessed directly, and after 4 weeks from discontinuing the CHX treatment^55^. Significant decreases in diversity were observed, with increased streptococci and with an increase in prevalence in genes encoding tetracycline efflux pumps. Moreover, through shotgun metagenomics sequencing from 179 patients that were either orally healthy, had active caries, or with periodontal disease, found 64 ARGs that imparted resistance to 36 antibiotics, particularly to beta-lactam, tetracycline and macrolide-lincosamide-streptogramin antibiotics. Notably, these corresponded to high phenotypic resistance^1^. A higher prevalence of these was found in healthy and caries active than in periodontally diseased individuals. Given that these bacteria can translocate and disseminate systemically, then the oral cavity represents an important consideration for AMR. A significant concern identified from the repeated use of CHX, where sequential passage of supragingival plaque treated with CHX over 10 days showed the ability to select bacteria with 2 to 4 x MIC. Alarmingly, concomitant resistance to antibiotics was observed, such as up to 12 x MIC for erythromycin and clindamycin^100^. Together, these CHX based studies indicate the unintended consequences of a key oral antiseptic; agents that indiscriminately kills or inhibit bacteria, viruses and fungi. This is the case for other antiseptics, where off-target effects are likely to lead to decreased colonisation resistance.

## (Anti- and Pro-) Biotic Interventions

Antibiotics are chemical agents produced by fungal or bacterial species designed to kill or inhibit the growth of bacteria. Scientific evidence implies that antibiotics have been unwittingly used for thousands of years, since the time of the ancient Egyptians. Traces of tetracyclines (a form of antibiotics) have been identified in the bones extracted from these ancient populations^101,102^. In the modern day, following the inception of the first identified antibiotic, Penicillin in 1918 by Sir Alexander Fleming, these antimicrobial agents are pivotal in managing bacterial infections across medical disciplines, including dentistry. In oral healthcare, antibiotics are often used for therapeutic interventions, particularly in severe infections such as periodontal abscesses. For many years, prophylactic antibiotics were also administered during dental therapy for preventing systemic complications such as IE in susceptible patients; however, in the UK such usage is seldom recommended in a routine manner anymore^103^.

Of the antibiotics used in oral care, many are broad-spectrum, with amoxicillin being the most common prescribed by dentists^104^, often to target mixed-species or undiagnosed infections due to its indiscriminatory antimicrobial activity. Some narrow-spectrum antibiotics, reserved for known pathogens, offer a focused approach that minimizes impact on non-target commensal bacteria. These provide important interventions for dental abscesses and severe cases of periodontitis, through targeting gram-negative anaerobic bacteria associated with such diseases. Nevertheless, metronidazole is the most common antibiotic prescribed in primary care suggestive that it is the often the go-to prescription for general dentists, regardless of disease severity^104^. Metronidazole is a prodrug, meaning it requires activation by bacterial species in anaerobic microenvironments. The activation occurs via reduction of the -NO_2_ group on the metronidazole molecule by microbial enzymes such as pyruvate:ferredoxin oxidoreductase to exert its antimicrobial effects^105^. This tends to only arise in oxygen deplete environments such as those deep within the periodontal pockets. Generation of nitrogen radicals produced during the reduction process leads to cytotoxicity exerted on targeted cells, including DNA damage through interference with the helical structure of the nucleic material^106,107^. Such reductase enzymatic systems are found in oral bacterial pathogens such as *P. gingivalis, P. intermedia, F. nucleatum*, and *T. forsythia*, but absent from aerobic commensal species, thus providing an ideal drug candidate for targeted therapy^108^. Further to its antimicrobial activity, metronidazole can also potentiate immune responses during anaerobic bacterial infections^109^.

**Fig. 2:**
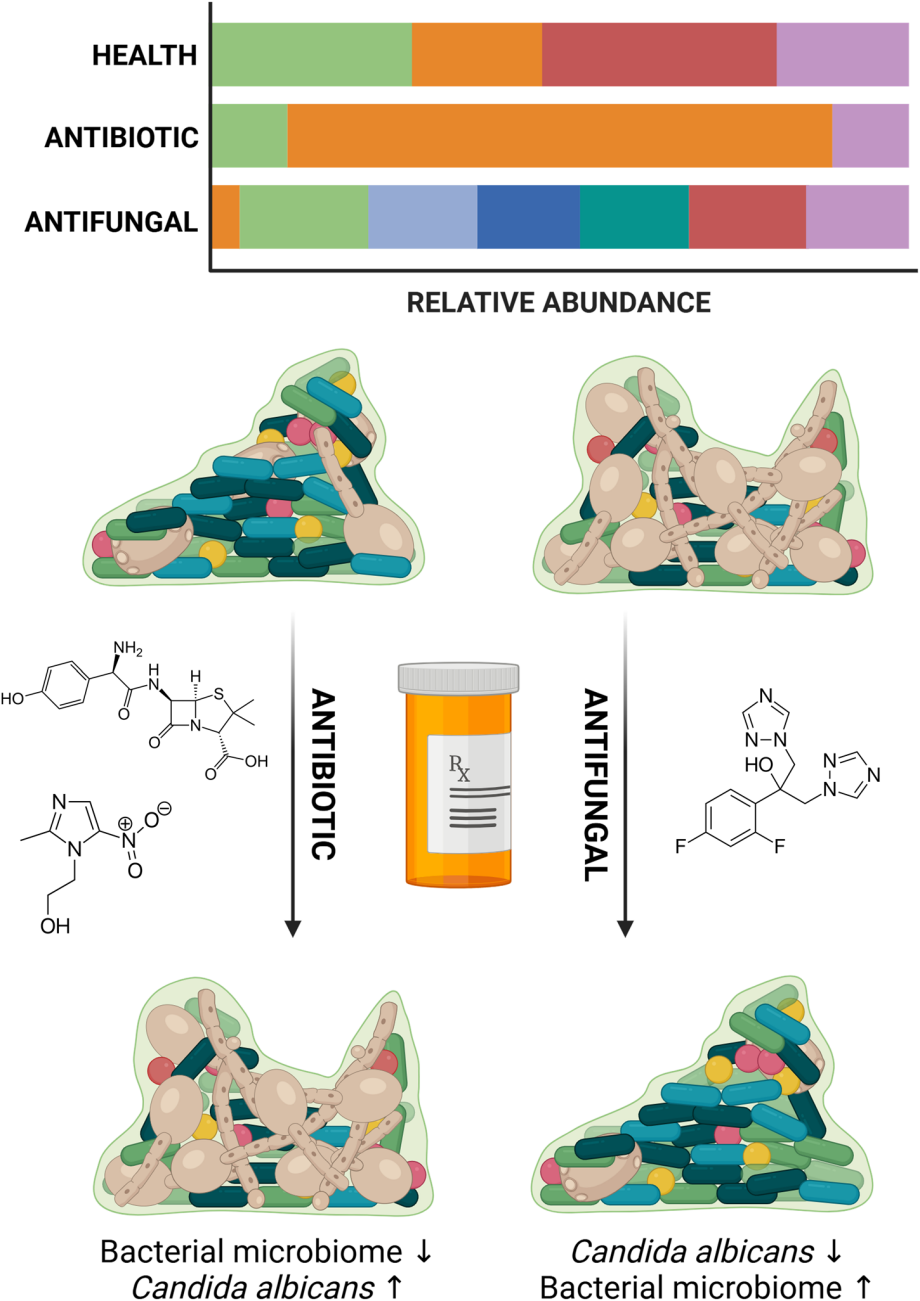
Antibiotic and antifungal interventions can modulate the oral microbiome. Antibiotic therapies such as amoxicillin and metronidazole are two of the most prescribed antibiotics in oral care. Although effective in combatting severe bacterial infections, can lead to resistance in some bacterial species, as well as a shift in the oral mycobiome. This is often associated with an overgrowth of *Candida albicans* (orange bar, in hypothetical graph), one of the main fungal colonizers of the oral cavity, increases that lead to hyphal formation and tissue invasion (e.g., oral thrush). Conversely, antifungal therapies such as fluconazole can reduce *C. albicans* numbers, which can lead to flourishment of bacterial populations within the ecological niches left behind. Figure created using BioRender.

Despite their regular prescription by dentists, the use of antibiotics comes with obvious controversy. Recent evidence suggests that antibiotics are frequently overused and misused in dental practice^110^. Over-prescription, especially in cases where non-antibiotic interventions like mechanical debridement or antiseptics might suffice, raises concerns about “collateral damage” to the existing oral microbiome and/or unnecessary systemic exposure (Fig. 2). With respect to the oral microbiome, several studies have documented reductions in pathogenic bioburden in plaque and improvement in clinical outcomes following systemic treatment with amoxicillin and/or metronidazole with or without mechanical therapy^111–116^. These findings underscore that, although antibiotics are not routinely indicated for periodontitis, they may be warranted in specific advanced cases. Worryingly though, extensive evidence exists that the oral microbiome can shift following antibiotic interventions: microbial profiles of the commensal populations can remain altered for months post-treatment following removal of antibiotic therapy^111,117,118^. These studies have shown increases in numbers of bacterial species such as *S. mutans* and *Haemophilus* spp., which are pathogens associated with dental caries and upper respiratory tract infections, respectively^111,118^. Such phenomena could be explained with the well-known idiom “robbing Peter to pay Paul”, whereby treating one disease could lead to the aetiology of another. Indeed, a recent systematic review identified positive correlations between periodontitis and respiratory complications such as chronic obstructive pulmonary diseases^119^.

To further compound potential complications, antibiotic-resistant bacterial species of the oral cavity have been reported. In periodontal pathogens, strains of *P. gingivalis*, *T. forsythia* and *A. actinomycetemcomitans* isolated from patients with periodontitis had varied levels of amoxicillin and metronidazole resistance^120^. Aligning with this, concerning trends in the US have emerged, whereby reports that *P. gingivalis* resistance is increasing in the last 25 years^121^. However, resistance has not only been reported in oral disease such as periodontitis. A recent study by Anderson et al.^1^ observed significantly more ARGs in the plaque of healthy individuals and dental caries patients than those with periodontitis. These genes included mef(A) and tet(M), conveying resistance to macrolides and tetracyclines, respectively^1^. Indeed, other oral colonizers, including commensals, also have different susceptibility profiles to common antibiotics used in dentistry. Amoxicillin-resistance in *Haemophilus* spp., *Streptococcus* spp., and *Veillonella* spp has been reported in children aged 4–5 years of age, with numbers of resistant isolates higher in those children that had received amoxicillin antibiotic therapy in early life^122^. Similar observations were present in adults, with the health and disease microbiome of the oral cavity acting as a reservoir for antibiotic resistance profiles within the microbiota^1^. As discussed above, the oral resistome develops in early life, and antibiotics can not only shape the oral microbiome, but systemic administration can affect the diversity of the gut. These dysbiotic changes can also result in increases to the resistance gene load in GI microbial populations, which can have severe implications that exude far beyond oral health^123,124^. To this end, shifts in the microbial composition at distal sites can also be a source by which the oral microbiome is disturbed leading to associated infections. Systemic antibiotic-induced gut dysbiosis following administration of a cocktail of cefoxitin, gentamicin, metronidazole, and vancomycin, induced long-term microbial changes in the gut microbiome of specific-pathogen free mice. These changes were associated with perturbation of the oral microbiota inducing onset of periodontitis, a phenomenon that was alleviated following transplantation with normal mice faeces^125^.

In vitro model systems have allowed scientists to study the impact of antibiotic therapy on complex microbial populations in a more controlled and reproducible manner. These populations are grown in the form of biofilms and can be treated with a range of concentrations of antibiotics. One particular study reported that low concentrations of ampicillin used against an ex vivo biofilm derived from human saliva promoted significant shifts in microbial taxons, leading to increases in biofilm viability. Increases in ARG abundance were observed in *Streptococcus* spp.^126^. This observation aligns with the recent notion that oral commensal streptococci can act as a sources for antimicrobial resistance within their genetic signatures, as discussed above^127,128^. In vitro studies have also investigated the phenomenon of persistence of key bacterial pathogens such as *P. gingivalis* to metronidazole, with resistant populations still maintaining their virulence mechanisms whilst enhancing stress responses^129,130^. Importantly, such persistence has been modelled in more complex, multi-species biofilms in vitro. Plaque, saliva or root canal derived biofilms in healthy or diseased patients have been grown and treated ex vivo, monitored following antibiotic interventions^131–133^. Such work has often highlighted donor variability in biofilm communities arising from differences in either their microbiomes and/or resistomes. One study even reported that results from ex vivo oral biofilm susceptibility testing was able to direct personalized therapies for patients with periodontitis in those that provided the samples for laboratory growth^132^.

Beyond antibiotic therapies, probiotics provide promising alternatives for controlling disease and creating a habitable microenvironment for commensal bacteria, yet one that is hostile to pathogens. Probiotics are live microorganisms that when consumed in adequate amounts, either in their viable or non-viable forms, confer health benefits on the host by balancing the microbial ecosystems in the body. They are often used to restore or maintain healthy microbiota in the GI tract, at the vaginal epithelium, and within the oral cavity. *Lactobacillus* spp. (e.g., *L. rhamnosus*, *L crispatus*) and *Bifidobacterium* spp. (e.g., *B. bifidum* and *B. longum*) have been used to control microbial populations within these different ecological niches. The mounting evidence for probiotic therapy to combat oral diseases is promising, with several recent systematic reviews and meta-analyses indicating positive outcomes in patients with oral diseases such as dental caries, periodontitis and halitosis^134–137^. Probiotic therapies hold many advantages over antibiotics, with one in particular being the ability to integrate probiotics into lozenges for localized targeting of the oral cavity. Studies have shown that probiotics delivered in this manner can improve gingival health, reduce plaque and enhance immunity^138,139^. Although dental material science continues to develop new and innovative methods for localized delivery of antimicrobials including antibiotics, we are still a long way from creating an alternative that bypasses the systemic implications associated with such therapy.

Probiotic treatments do not come without their own limitations. A particular challenge involves the establishment of the probiotic within the existing oral microbiome, with existing treatments often leading to a transient impact rather than a prolonged effect arising from colonisation. Although studies have shown that probiotic cocktails within lozenges can lead to oral colonization^138^, retention of the probiotic within the community often relapses soon after probiotic intake ceases^140–143^. Further complications can arise from strain variability, formulations, and delivery methods provide extreme challenges. As such no Food and Drug Administration (FDA) or Medicines and Healthcare products Regulatory Agency (MHRA)-approved probiotic therapies currently exist. Furthermore, treatment of one oral disease (e.g., periodontitis) through probiotic interventions may lead to adverse effects initiating onset of another disease in its place. For example, most probiotics produce lactic and acetic acids, which can create acidic microenvironments. In the gastrointestinal and vaginal health, acidification is not an issue given the pH is often below 4.5, however in the oral cavity, the microenvironment is more neutral with buffering effects of saliva maintaining a pH of around 6.0–7.5^144^. Low pH is often associated with dental caries arising from demineralization of the enamel. In vitro evidence has been shown that probiotic therapy can affect enamel microhardness, surface roughness and leaching of calcium and phosphorus, important elements that maintain healthy mineralisation^145^. It is noteworthy that not all probiotic strains cause such acidification. Several clinical studies have shown tha*t Streptococcus dentisani* 7746 has buffering capacities through ammonia production^146,147^. Similarly, in vitro studies have shown that *Rothia aeria* strain Ra9 significantly reduces pathogenic communities in periodontal biofilms, with only minor reductions in supernatant pH (to approximately pH 6.5)^148^. Nevertheless, careful monitoring and strain selection remain essential when considering probiotics for oral health applications.

## The Oral Mycobiome and Antifungals

The clinical management of fungi in the oral cavity is often a secondary consideration to bacterial diseases, where the primary concern is generalised plaque removal from enamel surfaces and the active physical removal of supra- and sub-gingival plaque. Even endodontic interventions and cleaning of prosthetic appliances is focused on bacterial disruption. *Candida* spp. are the most prolific and dominant of the fungi within the oropharynx, though there is growing evidence from the literature that diverse fungal floral (the oral mycobiome) exists in parallel. Here fungi are intertwined within a dense bacterial community, numerically outnumbered, and antagonised by the release of secondary metabolites. It is hardly surprising that this kingdom of life is seldom prioritised. Yet, careful mycological assessment indicates that yeasts are not only present but are also an important structural feature in polymicrobial communities^149^. Several studies have shown that *C. albicans* can provide a scaffold to singular and multi-bacterial communities found in the oral cavity^149–152^. Furthermore, *Candida* spp. such as *C. albicans* have been shown to provide metabolic support to bacterial species. For example, *C. albicans* can create anoxic conditions in an otherwise O_2_ rich environment to allow flourishment of anaerobic pathogens such as *P. gingivalis*^153–155^. Such microbial crosstalk has led to *C. albicans* being termed the “keystone commensal” in the healthy oral cavity, due to its ecological impact on the proximal community and immediate microenvironment^149,156^. The unnecessary and overuse of anti-bacterial agents and physical plaque removal strategies described above, carves out a niche for fungi to thrive and dominate some oral environments, albeit in an opportunistic manner to the genetically predisposed or with underlying comorbidities (Fig. 2).

Oral candidal infections are principally superficial infections^157^, generally caused by an overgrowth of *C. albicans*. Other non-*albicans* species, such as *Candida glabrata, Candida dubliniensis, Candida krusei, Candida parapsilosis, Candida stellatoidea, Candida tropicalis* and *Candida guilliermondii* are also contributing causative agents. A range of diseases are associated with these yeasts, including pseudomembranous candidosis, angular chelitis, chronic atrophic (denture associated) and chronic hyperplastic candidosis. In these diseases yeasts and hyphae (filamentous forms) coaggregate and become intertwined upon mucosal surfaces to give the clinical appearance of thick white plaques (biofilms) that sit externally to the mucosa. They are covered by a beta-glucans matrix, which is a glue-like material that supports architecture and is a key contributor to antimicrobial tolerance within an interkingdom biofilm^158^. Crucially, *Candida* species do not live in solitude, and instead play an important role within interkingdom biofilms^159^. They are generally isolated in less that 2 logs to that of bacteria species^160^, but occupy a similar biovolume. This disproportionate content is observed in other prevalent oral chronic biofilm diseases, e.g., dental caries and periodontal diseases, both of which are considered primarily bacterial driven diseases^161,162^. Notably, it has been demonstrated that *Staphylococcus aureus* is able to sequester and cover themselves in *C. albicans* glucans within interkingdom biofilms, and that this confers vancomycin resistance^163^. This is important when we consider that *C. albicans* can facilitate systemic bacteriaemia by *S. aureus*^164^. Miconazole is often used to manage angular chelitis, which is often characterised by mixed *C. albicans* and *S. aureus* infections. While used topically at the angles of the mouth it has been shown to influence both fungi and bacteria, largely through non-specific membrane disruption, the production of reactive oxygen species and enzymatic inhibition^165^. Membrane disruption is a cornerstone of antiseptics for the treatment of oral candidiasis, with first line therapy usually involving the use of CHX to support the clearance of yeasts. As eluded to above, this can have affect the microbial community. Therefore, targeted antifungal agents appear a more beneficial strategy.

The azole fluconazole (FLU) is arguably the most heavily used antifungal agent in the oral cavity. It inhibits ergosterol biosynthesis through targeting the 14-lanosterol demethylase enzyme pathway. This prevents ergosterol biosynthesis, which in turn leads to lipid membrane destabilisation and cell death. Mutations in the CaCYP51A gene are known to be associated with resistance. This is a fungistatic antifungal, and like other triazoles (e.g., itraconazole), it is used to manage a range of chronic oral candidal conditions^166^. Given the specificity of its mode of action, then there is no reason to speculate that it could influence the oral microbiome, though this appears not to be the case. Several in vitro studies indicate that FLU has the capacity to inhibit planktonic bacterial species. For example Dornelas-Figueira et al.^166^, reported fluconazole sensitivity in *Streptococcus mitis, Escherichia coli* and *Granulicatella adiacens*. There is evidence that some mycobacteria and streptomycetes species encode cytochrome P450 mono-oxygenases (P450s), which are homologues of 14α-sterol demethylases^167^. These are essential for viability and/or growth of both bacterial genera. Recent in vivo studies have shown that FLU- exacerbated inflammation in an induced asthma murine model, with exhibited higher Bacteroidota levels, lower Firmicutes, and reduced bacterial abundance^168^. Interestingly, in the GI tract it has been shown that FLU treated mice aggravate sepsis through eradication of gastrointestinal fungi. Here, the fungi inhibit Gasdermin D cleavage, which is a requisite for endotoxin release^169^. Together these data highlight the potential for azole antifungals to exhibit wider reaching effects beyond fungi that influence microbiome stability.

Amphotericin B (AMB) is a polyene, and these are thought to insert into the lipid membrane adjacent to ergosterol^170^, which in turn destabilises the cell membrane by forming pores and enabling cellular lysis^171^. Moreover, oxidative stress induction is also thought to additionally contribute to its effectiveness as a fungicidal agent^172^. Resistance is rare due to its membrane-based target, but in some cases alterations to sterols and anti-oxidative stress mechanisms can protect the cell. Cell wall changes in the form of enhanced 1,3-alpha- and 1,3-beta-glucans and sphingolipids have also been shown to correlate with AMB resistance^173^. Recent work has shown that in interkingdom populations the presence of *Pseudomonas aeruginosa* impacts oxidative stress pathways, and that phenazines may be an important driver of this. The mitochondrial superoxide dismutase SOD2 is significantly down-regulated in the presence of *P. aeruginosa*, which in turn sensitises *C. albicans* to the effects of AMB^174^. This indicates that microbial complexity can positively impact the effects of antifungal drugs. Conversely, it has been revealed from molecular docking studies that AMB mode of action analysis targeting penicillin binding protein 2a (PBP 2a protein), a target of β-lactam drugs^175^. Indeed, these studies revealed activity against MRSA isolates in the range 8–32 ug/ml. While crude in nature, these assays reveal the broader antibacterial impact of AMB. We can hypothesise that the use of AMB (nystatin) for local delivery in the oral cavity has the potential to destabilise other Gram-positive bacteria and negatively influence community structure. In our own experience we have shown that both AMB and FLU can cause profound effects to an experimental ex vivo oral microbiome (unpublished data), indicating that it has an influence beyond *S. aureus*.

*C. albicans* and other yeasts are known to release farnesol and tyrosol, quorum sensing molecules associated with yeast to hyphae morphological regulation^176–178^. Research activities have been extensive in investigating how farnesol in particular influences bacterial behaviour. Animal studies have shown that tt-farnesol was able to modestly reduce mutans streptococci^179^. It was also demonstrated that this also inhibited *S. mutans* polysaccharide production and biomass accumulation^180^. The effects of farnesol extend beyond *Streptococcus* spp, with anti-bacterial and anti-biofilm activity reported against *S. aureus* and ESKAPE pathogens^181–183^. What this means is that *Candida* spp. within the oral cavity exude their own capability to influence the oral microbiome, and fluctuations to the balance of yeasts can negatively impact the development and function of the dominant bacterial communities. Therefore, targeted antifungal therapies may result in undesired consequences with respect to natural control of caries and periodontal pathogens. Together, these studies highlight that we need to consider fungi more carefully when prescribing antibiotics and antifungals, as tipping the balance between bacteria and fungi can have profound implications.

## Concluding Remarks

Together, the experimental and clinical studies outlined above emphasizes the complex and sensitive nature that exists within the oral microbiome. As we try and control plaque and prevent oral diseases then there are unintended consequences. The “butterfly effect” induced by alterations in the different parts within the biogeography of the oral cavity has the potential to drive significant and uncontrolled collateral damage to the distal oral and systemic sites. Whether this is through diet induced bacterial dysbiosis, evolution of drug resistance phenotypes or overgrowth of yeasts within complex microbiomes, these all have the potential to tip the balance to a dysbiotic state. It is difficult to predict from patient-centred or clinically managed plaque control whether the outcomes will be positive or negative. However, perhaps the use of bioinformatic and machine learning approaches will offer some degree of predictability that will in turn allow a more targeted and nuanced tactics to manage oral health. To this end, recent research has highlighted artificial intelligence-based models are starting to utilise host metadata to allow for prediction of systemic diseases with higher accuracies^184,185^, simply through profiling of a patients oral microbiome^186^.

## Data Availability

No datasets were generated or analysed during the current study.
